# Pollution Emissions, Environmental Policy, and Marginal Abatement Costs

**DOI:** 10.3390/ijerph14121509

**Published:** 2017-12-05

**Authors:** Ling-Yun He, Jia-Jia Ou

**Affiliations:** 1 College of Economics and Management, China Agricultural University, Beijing 100083, China; lyhe@amss.ac.cn; 2 Institute of Resource, Environment & Sustainable Development Research, School of Economics, Jinan University, Guangzhou 510632, China; 3 School of Economics and Management, Nanjing University of Information Science and Technology, Nanjing 210044, China

**Keywords:** SO_2_ emissions, marginal abatement costs, shadow prices

## Abstract

Pollution emissions impose serious social negative externalities, especially in terms of public health. To reduce pollution emissions cost-effectively, the marginal abatement costs (MACs) of pollution emissions must be determined. Since the industrial sectors are the essential pillars of China’s economic growth, as well as leading energy consumers and sulfur dioxide (SO_2_) emitters, estimating MACs of SO_2_ emissions at the industrial level can provide valuable information for all abatement efforts. This paper tries to address the critical and essential issue in pollution abatement: How do we determine the MACs of pollution emissions in China? This paper first quantifies the SO_2_ emission contribution of different industrial sectors in the Chinese economy by an Input-Output method and then estimates MACs of SO_2_ for industrial sectors at the national level, provincial level, and sectoral level by the shadow price theory. Our results show that six sectors (e.g., the Mining and Washing of Coal sector) should be covered in the Chinese pollution emission trading system. We have also found that the lowest SO_2_ shadow price is 2000 Yuan/ton at the national level, and that shadow prices should be set differently at the provincial level. Our empirical study has several important policy implications, e.g., the estimated MACs may be used as a pricing benchmark through emission allowance allocation. In this paper, the MACs of industrial sectors are calculated from the national, provincial and sectoral levels; therefore, we provide an efficient framework to track the complex relationship between sectors and provinces.

## 1. Introduction

Ambient air quality in many Chinese cities exceeds both national standards and international guidelines [1,2]. Sulfur dioxide (SO_2_) emission is one of the most serious air pollution problems in China, causing a series of environmental problems and health issues [3,4,5,6,7,8]. As the largest SO_2_ emitter globally (According to statistics, national energy-related SO_2_ emissions in China were several times than those in other countries from 2011 to 2013. (Source: National Bureau of Statistics of the People’s Republic of China, World Bank and The Statistics Portal)), China has also actively undertaken this duty of its mitigation, and implemented many policies and programs, such as 10th Five-Year (2001–2005) and 11th Five-Year (2006–2010) Plans, which are a series of social and economic development initiatives, and contain detailed economic development guidelines for all its regions. The SO_2_ reduction goal was to reduce SO_2_ emissions 10% below the levels of the year 2000 according to the 10th Five-Year Plan. In the 11th Five-Year Plan, the goal of abating SO_2_ was to reduce the emissions 10% below the levels of the year 2005. The 12th Five-Year (2011–2015) and 13th Five-Year (2016–2020) Plans (For the details please visit the website http://www.gov.cn), are also ambitious to reduce SO_2_ emissions and improve air quality. However, China is encountering great pressure to implement emission reduction because it is undergoing a rapid industrialization process with massive energy consumption and thriving industrial production [9,10]. The coal-type consumption share is the largest due to China’s energy endowment [11]. Such a coal-dominated energy structure in China’s industrial sectors has caused a large amount of SO_2_ emissions, and thus the environmental deterioration for a long time. Thus, how to solve environmental problems cost-effectively in China’s industrial sectors attracts many attentions from the policy makers and the academia as well.

Air pollution is a typical example of “Negative Externalities” in economics. The key to solving the environmental problem in production is how to make firms take into account the cost of the negative externality. Generally speaking, there are two approaches to reduce emissions in economic theories. One is the Pigouvian tax, designed to correct negative externalities, that imposes costs (e.g., pollution taxes) on emitters. A pollution tax is a policy measure by government as it has relatively low transaction costs associated with implementation. But, it’s impossible for the government to know all information affecting individuals to set the optimal tax rate. Thus, the government starts to pay more attention to the other approach [12,13,14,15,16]: the market approach (for example, the emission trading systems, based on Coase [17]), which is characterized by cost-effectiveness, flexibility, certainty about quantity, and minimizing risk. Later, many countries established active trading programs in air pollutants to resolve environmental problems, such as the SO_2_ trading market in America, and the carbon trading system in the Europe.

Since 2007, the State Council of the People’s Republic of China (SCPRC) has started some pilot emission trading programs in several provinces of China. Then the SCPRC released “Guiding opinions on further promoting the pilot work of paid use and trading of emission rights” in 2014 [18], which means that a national-level pollution emission trading system (ETS) will be established in China. The implementation of total emission amount control is the premise of these pilot projects. According to the SCPRC, these projects in China should adopt quota selling or auction to assign pollution rights. The price of quota selling can be determined by the local pollution control costs, environmental resource scarcity, economic development level, etc. The reserve price in an auction can refer to the standard of quota selling. The polluters shall obtain the pollution rights by trading pollution emission allowances. However, Wang and Zhang [19] point out that these pilot projects in China are controlled by the government and lack of price formation mechanism. Pollution pricing is one of the important functions of the pollution ETS. In an effective pollution pricing mechanism, tradable allowances can create an incentive for polluters to determine the most cost-effective approach to reducing pollution [20]. In this context, it is crucial for the academia and policy makers to understand the operation of the emerging pollution ETS in China.

Our paper aims to explore a key issue relevant to China’s pollution ETS: the marginal abatement costs (MACs) of SO_2_ emissions. Estimating the marginal abatement costs (MACs) can provide valuable information for the policy makers to improve the operating rules of ETS, especially pollution pricing. The pollution ETS transforms SO_2_ emission allowances into a new type of tradable financial product. Firms can make decisions by comparing market prices of allowances with MACs. The sectors with low MACs (compared with the market price) may offer unused allowances for sale and owners with high MACs may shop for sale if they hold insufficient allowances to cover their planned emissions. Only when a rational pricing mechanism is established, the government can truly achieve the abatement goals by transferring the pollution abatement tasks from the production units with higher MACs to the ones with lower MACs. More importantly, if this SO_2_ trading market works as planned, one should see the cost of reducing additional amounts of SO_2_, which is equal to MACs [21,22]. Therefore, the MACs should be used as a reference for pollution pricing through emission allowance allocation.

In summary, the following key issues should be addressed: At the first phase of the trading market, which sector(s) should be mandatory for participation? What are the MACs of SO_2_ emission allowances? At first it is essential to quantify the emission contribution of different industrial sectors in the Chinese economy. By doing so, major emitters can be identified; and policy makers may know what industrial sectors should be covered in the Chinese pollution ETS. Then this paper estimates marginal abatement costs of China’s industrial sectors at the national, provincial, and sectoral levels. Once the MACs of pollution emission allowances at these three perspectives are estimated, our empirical study could provide key information for the policy makers to formulate more appropriate pollution abatement policies in the operation of pollution ETS.

To estimate the emission contribution of SO_2_, this paper applies the input-output (I-O) method to distinguish the sectoral emissions and identify the shares of individual industries [8,23]. Multiple methods have been used to estimate the MACs of harmful emissions, such as Cost-Benefit Analysis, Dynamic Optimization Model, Input-Output Analysis, Computational General Equilibrium Model, Integrated Assessment Model and Shadow Price Approach [22,24,25,26]. The concept of shadow price is used widely to estimate marginal abatement costs [21,27,28,29,30]. The shadow prices of SO_2_ emissions may be interpreted as the opportunity cost of an incremental SO_2_ reduction in terms of giving up good outputs in a production process. Many scholars have begun to study the shadow prices. For example, Swinton [21] provides an estimation of shadow prices of SO_2_ abatement using the output distance function approach for coal-burning electric plants. Mekaroonreung and Johnson [29] formulate a convex non-parametric least square quadratic optimization problem to estimate a frontier production function and apply the method to estimate shadow prices of SO_2_ and NO_x_ generated by the U.S. coal power plants. He and Chen [31] introduce a dynamic optimization method to estimate shadow prices of water right. These methods only calculate the shadow price in a specific industry or national level and may not be useful in calculating shadow prices in provincial level. This paper calculates the shadow prices of SO_2_ emission allowances by a pricing model based on the shadow price theory, which is very flexible in the level of application and can be applied to the cases of sectors, regions, or even the whole country. As discussed earlier, to realize the overall goal, the firm can sell excess SO_2_ emission allowances with a higher price (e.g., its shadow price) than the market price under the premise that the firm will have sufficient SO_2_ emission allowances. With the sale of superfluous SO_2_ emission allowances in the market, the market will reach an equilibrium until the shadow price is equal to the market price. Thus, this paper calculates the shadow price of SO_2_ emission allowances based on the principles of operational research, reflecting the intrinsic value and marginal cost of allowances. Using this model combined with the I-O method, the paper can estimate shadow prices of industrial sectors from the national, provincial and sectoral levels; therefore, it offers an efficient way to track the complex relationship between sectors and provinces and can compare the shadow prices of different provinces and sectors in a consistent statistical caliber.

This paper is organized as follows. Section 2 introduces the structure of an integrated assessment framework of shadow price model and the SO_2_ emission calculation model on energy consumption. Section 3 shows the data source in this paper. Section 4 presents the results and discussions. Finally, Section 5 summarizes our results and provides some policy implications.

## 2. Methodology

### 2.1. The Estimation of Sulfur Dioxide Emissions

The paper estimates SO_2_ emissions of China and different industries, and identifies the shares of individual industries. If the shares of individual industries can be identified, pertinent policies can be made in the pollution ETS, e.g., the main emitters should be covered in the first phase of the pollution ETS. At first, this paper applies the method provided by the Intergovernmental Panel on Climate Change to evaluate the emissions of SO_2_ based on energy consumption and then calculates the SO_2_ emissions of different industries, applying the method given by Gemechu et al. [23] and He and Ou [8].

First, the paper uses Equation (1) to estimate the sulfur dioxide emissions from different types of energy consumption. Energy categories include Coals, Gas and Refined Petroleum products and Coke, which can be regarded as the productions of energy-producing sectors in the Input-Output table. For example, Coals contain raw coal, cleaned coal, other washed coal, and briquettes, which can be seen in Table 1. The method of calculating energy consumption, based on the energy balance sheet, can avoid the omission or double counting of the secondary energy consumption due to its direct reflection on the input and output of energy production. Thus, this paper doesn’t consider Heat and Electricity because the paper has already regarded them as output of transformation. In summary, this paper gets the total SO_2_ emissions *Q* in energy consumption by adding the emissions of different energy types.

(1) $$Q_{j}=\left(Z_{j}+S_{j}\right)\cdot K_{j}\cdot \alpha_{j}$$

where $Q_{j}$ refers to SO_2_ emissions for different kinds of energy *j*; *Z* refers to the final fuel consumption; *S* refers to the fuel consumption for thermal power and heating supply; *K* refers to emission factors of SO_2_; α refers to oxidation factors, which is assumed to be 1. Here, $K_{j}$ values come from Kato and Akimoto [32]; $Z_{j}$ and $S_{j}$ values come from the Energy Balance Tables of China (physical quantity) in 2012.

Second, this paper estimates the emissions of SO_2_ on different industries in an Input-Output method. The paper uses the Input and Output Tables in 2012, which contain 41 sectors and industries. As you can see, the paper has already got the emissions of SO_2_ on different energy categories, which is the productions of four energy-producing sectors. Energy-producing sectors contain Mining and Washing of Coal (MWC), Extraction of Crude Petroleum and Natural Gas (ECPNG), Manufacture of Refined Petroleum, Coke Products, Processing of Nuclear Fuel (MRPCPPNF), and Production and Distribution of Water (PDW). Furthermore, this paper unites ECPNG and PDW into a new sector, called Crude Oil and Gas (COAG). The three new sectors can produce corresponding energy categories. Once know the total use of each new sector, this paper can calculate the emissions of unit input, called energy emission factors on using (see in Equation (2)).

(2) $$w_{e_{s}}=\frac{Q_{d}}{I_{s}+Y_{s}}$$

where $w_{e_{s}}$ refers to energy emission factor on using for energy produce sector *s*; $I_{s}$ refers to the total intermediate use, which is equal to the intermediate input; $Y_{s}$ refers to the total final use; $Q_{d}$ refers to the emissions of the energy production *d* (Coals, Gas or Refined Petroleum products and Coke). For example, the emissions of Coals are calculated by adding the emissions of raw coal, cleaned coal, other washed coal, and briquettes. Here, the values of $I_{s}$, $Y_{s}$ come from the Input and Output Tables in 2012.

Above all, once know different industries’ input in the three sectors, we can calculate the sulfur dioxide emission in different industries. The equation is the following one. Therefore, the paper calculates the SO_2_ emission of different industries in the whole China and different provinces by using national and provincial Input-Output tables.

(3) $$Q_{i}=\sum_{s=1}^{3}\left(w_{e_{s}}\cdot I_{si}\right)$$

where $Q_{i}$ refers to SO_2_ emission for industry *i*; $I_{si}$ refers to different industries’ input on energy produce sector *s*.

### 2.2. Shadow Prices of Sulfur Dioxide Emissions

This paper uses the shadow price model in pricing SO_2_ emission allowances, which reflects the intrinsic value of SO_2_ emission allowances and can be regarded as the marginal abatement cost. The sulfur dioxide emission allowance is a scarce resource. The shadow price of SO_2_ emission allowances is treated as the premise price that a country or region (or sector) optimally uses SO_2_ emission allowances to pay for. The assumptions of the shadow price model are shown as follows:

**Assumption** **1.** Assuming that there is a given area where energy-saving, emission reduction targets, and emission reduction technology determine the region’s total SO_2_ emissions Q in a year, i industries (or companies) participate in emission allowance trading (i = 1, 2, …, n).

**Assumption** **2.** Assuming SO_2_ emissions are proportional to the output value, because the burning of fossil fuels is the main source of SO_2_. In a given period of time during which the level of production technology remains unchanged, fossil energy consumption is proportional to production activities.

**Assumption** **3.** Assuming the initial allocation of emission allowances is based on paid distribution; the objective function is to maximize the company’s profit; the constraint is the total amount of SO_2_ emissions.

According to the above assumptions, we can get the equations:

(4) $$Q=\sum_{i=1}^{n}Q_{i}$$

where $Q_{i}$ refers to SO_2_ emissions in industry *i*; and

(5) $$Q_{i}=r_{i}\times q_{i}$$

where $q_{i}$ refers to the annual output value of industry *i*; $r_{i}$ refers to the average proportion coefficient of the output value according to Assumption 2, negatively correlated with internal pollution governance‘s inputs [33].

Then, the problem is converted to linear programming to solve the following equation on the basis of Assumption 3:

(6) $$MaxB=\sum_{i=1}^{n}\left(B_{i}\times q_{i}\right)=\sum_{i=1}^{n}\left(B_{i}\times \left(Q_{i}÷r_{i}\right)\right)=\sum_{i=1}^{n}\left(\left(B_{i}÷r_{i}\right)\times Q_{i}\right)\;s.t.\sum_{i=1}^{n}\left(Q_{i}\leq Q\right)$$

where $B_{i}$ refers to the unit output revenue of this *i* industry. Firm’s objective function is to maximize its profit, which is equal to $B_{i}$ multiplied by $q_{i}$ in this equation. And then this paper uses Equations (4) and (5) to derive the last equal sign.

Using the Lagrange multiplier method to solve the linear programming problem, we can derive the Equation (7).

(7) $$L=\sum_{i=1}^{n}\left(\left(B_{i}\times Q_{i}÷r_{i}\right)+\lambda \left(Q-\sum_{i=1}^{n}Q_{i}\right)\right)$$

where λ is as the Lagrange multiplier.

Based on the above equations, the first order partial derivative of $Q_{i}$ can be obtained in Equations (8) and (9).

(8) $$\frac{\partial L}{\partial Q_{i}}=n\times \left(B_{i}÷r_{i}-\lambda \right)=0$$

(9) $$\lambda =B_{i}÷r_{i}$$

Hence λ is the shadow price of unit emission allowance, which stands for the equilibrium permit price emerging from a cap-and-trade system. Using this model, we can calculate shadow prices of SO_2_ emission allowances in China.

## 3. Data

In the shadow price model, the profit of unit output value is used to replace the income generated by the unit output, using $B_{i}$ as a representative. In this paper, the profit of unit output value is the ratio of the total profit and the total output value in a particular period. Besides, in this paper, the formula for the proportion coefficient of SO_2_ emissions on the unit production scale, using $r_{i}$ as a representative, is the amount of emissions divided by the output value.

Data sources are “China Statistical Yearbook” (2012) and “Statistical Yearbook” of different provinces (2012), from which we select the total industrial output value and the total profits of various industries; “Input-Output Tables of China” (2012) and “China Energy Statistical Yearbook” (2012), from which we select the main energy consumption amount of the various industries.

The calculation of the SO_2_ emissions from various industries is based on the Equation (3) and the SO_2_ emission allowances’ shadow prices of different industries in China and 28 provinces for 2012 are calculated based on Equation (9). Due to lack of data it does not include Hong Kong, Macao and Taiwan, Gansu, Inner Mongolia and Tibet.

## 4. Results and Discussions

In this section, the main findings of the research are presented. The section begins with Table 2 that shows Chinese industrial sectors in the I-O tables. National Development and Reform Commission (NDRC) issues the notice of building the national carbon emissions trading market in 2017. There are already some regulations for the national carbon emission trading market in 2017, which may guide the pollution emission trading market. According to NDRC, national carbon emissions trading will cover the industries including petrochemical industry, building materials industry, building materials industry, iron and steel industry, non-ferrous industry, paper and pulp industry, electric industry, and aviation industry. The industrial sectors contained in our calculation cover these above industries.

This paper focuses on the shadow prices of SO_2_ emission allowances at the national level, provincial level, and sectoral level to explore the pollution emission trading mechanism. The Chinese pollution ETS should follow three fundamental principles, i.e., reducing pollution abatement costs through a government-guided market system, aiming at controlling pollution emissions, and focusing on industries with high emissions. The intention in this paper is to determine the price of pollution and let firms to bear the cost. Our paper pays more attention to the heavy-polluting industries which produce relatively more SO_2_ emissions and have lower shadow prices. If an industry doesn’t pollute the environment seriously, we shouldn’t consider it into the first phase of the market. Based on this consideration, this paper only selects the polluting industries whose SO_2_ emissions are over 1% of the total emissions in a given area. Thus, the paper only shows the shadow prices of polluting industries in the following text.

### 4.1. The Shadow Prices of Sulfur Dioxide Emission Allowances in China

**Figure 1:**
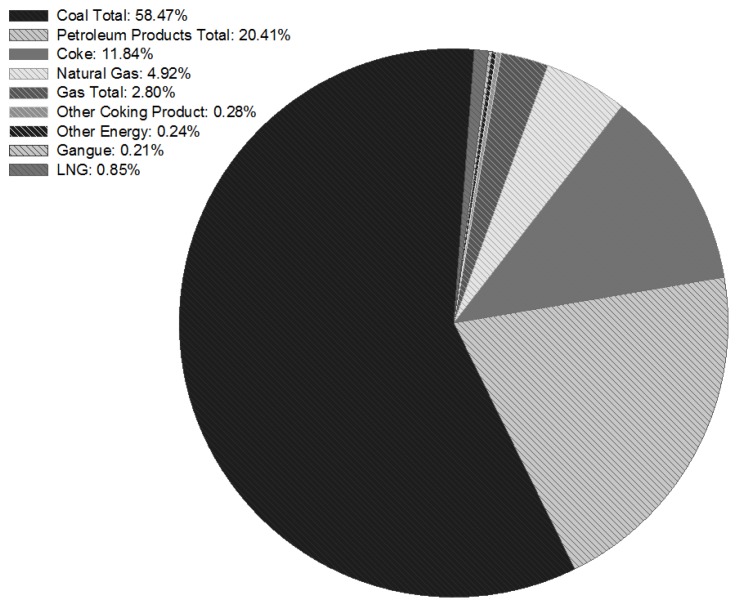
The energy comsumption ration of China in 2012.

According to the national energy balance sheet, Chinese energy consumption ratio in 2012 is shown in Figure 1. It can be seen that Coal Total is the primary energy source, accounting for 58.47% of total energy consumption. Petroleum Products Total accounts for 20.41%, Coke accounts for 11.84%, Natural gas accounts for 4.92%, and other energies account for a relatively small proportion. Fossil fuels have been playing the dominant role and the coal-type consumption share is the largest due to China’s energy endowment. China’s coal-dominated energy mix poses special difficulties to emission reduction. Given consideration to both China’s economic development and SO_2_ reduction targets, estimating MACs of SO_2_ emissions is the key aspect for the emission mitigation in China’s industrial sector or even the whole China.

**Figure 2:**
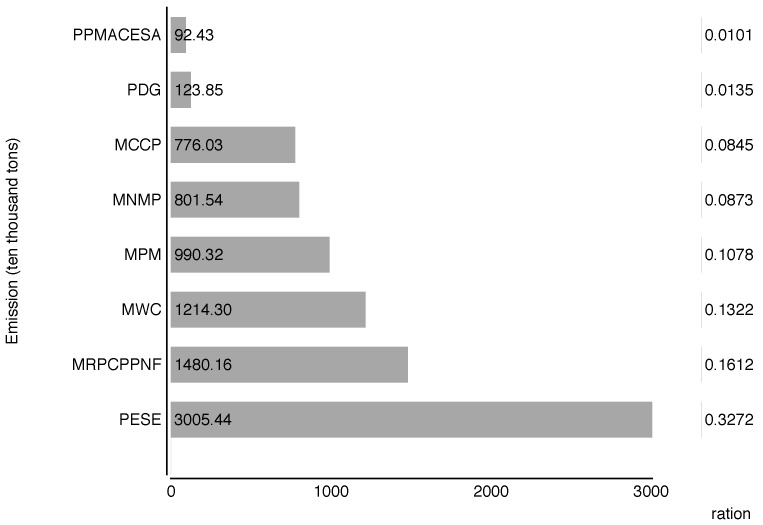
The main industries of releasing SO_2_ emission.

The polluting industries are shown in Figure 2, which in total cover over 70% of the national SO_2_ emissions. PESE, MRPCPPNF, MWC, MPM, MNMP, MCCP, PDG, and PPMACESA are the main emitters of China, which contain the industries covered by national carbon emission trading market. To abate national SO_2_ emissions, the policy makers may need to concentrate heavily on the key industries, especially those major emitters.

There are large differences in the shadow prices of polluting industries. As Table 3 indicates, the lowest shadow price is 0.20 ten thousand Yuan/ton (ttY/t) in MRPCPPNF; the highest shadow price is 19.09 ttY/t in PPMACESA. The profit of unit output value of MRPCPPNF is small, but relatively more SO_2_ emissions, therefore the marginal cost of further reduction is very small. The profit of PPMACESA is big, but relatively less emissions, so its shadow price is very high. Although PSES is the biggest emitter of sulfur dioxide emissions at the national level, the marginal cost of further reduction is lower than other industries, except for MRPCPPNF. Thus, PESE and MRPCPPNF have the bigger potential in reducing pollution. MCCP and PPMACESA may be buyers in the pollution trading market because of their higher gains of using an additional unit of allowance outweigh the cost.

### 4.2. The Shadow Prices of Sulfur Dioxide Emission Allowances of Different Provinces

China has a vast territory; the economic development level and pattern of industrialization and urbanization significantly differ across provinces. It is necessary to calculate the shadow prices of SO_2_ at the provincial level. Since 2007, the Ministry of finance, Ministry of environmental protection, and Development and Reform Commission have approved 11 local pilot pollution emission trading programs [34]. In this section, the paper only shows the shadow prices of pilot provinces, including Jiangsu, Zhejiang, Hunan, Hubei, Henan, Hebei, Shanxi, and Shaanxi (see Table 4).

At provincial level, the variation in different shadow price estimates could help our country identify a least-cost strategy for SO_2_ emission abatement. The overall weighted average of shadow prices (OWASPs), which is weighted by the SO_2_ emissions’ shares of individual industries at provincial level, of Jiangsu, Zhejiang, Hunan, Hubei, Henan, Hebei, Shanxi and Shaanxi are 3.70, 2.37, 1.99, 5.93, 2.32, 0.88, 1.19 and 2.44 ttY/t, respectively. The highest OWASPs is 3.44 ttY/t in Shaanxi, showing that the potential for reducing emissions in Shaanxi is relatively small. The lowest OWASPs is 0.88 ttY/t in Hebei, showing that Hebei has a greater potential in emission reduction.

Jiangsu: The information of shadow prices in Jiangsu is shown in Table 4. The lowest shadow price is 0.68 ttY/t in PSES because the profit of PSES in Jiangsu province is relatively small compared with its emission. Thus, it’s necessary for PSES to take measures to control its emission. The highest shadow price is 28.22 ttY/t in CN because of the relatively big profit. From the Input-Output table of Jiangsu province, the total output value ratio of CN is 6.64%, which is higher than most industries. Thus, CN in Jiangsu could be a buyer in the market. The shadow prices of MCCP and CN are higher than PESE, MRPCPPNF, MWC, MPM, PPMACESA, and MNMP.

Zhejiang: As you can see in Table 5, this paper shows the industries whose SO_2_ emissions are over 1% of the whole province. In these industries, the lowest shadow price is 0.90 ttY/t in PESE, and the highest price is 17.97 ttY/t in MCCP. Although the shadow price in MWC is only 0.37 ttY/t, the SO_2_ emissions of MWC are below 1% of the total emission. Thus, the paper doesn’t show it in the Table 4. Besides, the profit of MWC in Zhejiang province is relatively small. The same is MRPCPPNF.

Hunan: As the chart shows, the lowest shadow price is −0.41 ttY/t in MRPCPPNF. The profit of MRPCPPNF in Hunan is negative. But MRPCPPNF discharges more SO_2_ emissions, the government should pay more attention to MRPCPPNF. The highest shadow price is 11.78 ttY/t in MCCP. The industries of low SO_2_ shadow prices are PSES, MWC, and MNMP respectively.

Hubei: MRPCPPNF has a negative profit in 2012, so the shadow price is negative. The emission in MRPCPPNF is big. The government should pay more attention to MRPCPPNF. The shadow price in CN is very high, up to 25.91 ttY/t, with relatively higher profit and less emission. The shadow price of PSES is 1.02 ttY/t. The shadow price of MWC is the lowest. MNMP, MCCP and MPM also should be highlighted.

Henan: As shown in Table 4, the lowest shadow price is 0.10 ttY/t in PSES. Besides, the SO_2_ emission of PSES is the lowest. The second-lowest shadow price is 0.57 ttY/t in MRPCPPNF because of the big emission amount and low profit. The highest shadow price of MNMP is 29.33 ttY/t in Henan. Therefore, the government should pay more attention to PSES, MRPCPPNF, MWC, MNMP, MPM, and MCCP.

Hebei: The most prominent five industries are PESE, MRPCPPNF, MWC, MPM, and MNMP. The shadow prices of PESE, MRPCPPNF, MWC, MPM, and MNMP are 0.64, 0.06, 5.51, 1.80, and 2.83 ttY/t, respectively.

Shanxi: As Table 4 describes, the amount of sulfur dioxide emission from the industrial sectors is not large, because our calculation of sulfur dioxide emission is based on the I-O table. Shanxi is rich in mineral resources and holds the very great proportion reserves in the national coal mine. Most of Shanxi cities are the mining cities. The productions of MWC are sent out to other provinces. Thus, the industries’ energy using releases a small number of emissions. MRPCPPNF has negative profits, so its shadow price is negative. There is little difference between SO_2_ emission of MWC and PSES, but the profit of MWC is higher than PESE. According to the emissions and shadow prices, the government should take more heavy environmental regulations on MRPCPPNF, MWC, PESE, and MPM.

Shaanxi: The shadow prices of Shaanxi are not very small. The lowest shadow price is 2.44 ttY/t in PESE. The shadow price of CN is the highest. PESE, MRPCPPNF, and MNMP have a greater potential in emission reduction.

### 4.3. The Shadow Price of Sulfur Dioxide Emission Allowances in Key Industries

According to the results from the above pilot provinces, this paper regards MWC, MRPCPPNF, MCCP, MNMP, MPM and PESE as key industries. The shadow prices of these industries are shown in Table 5.

The variation in the estimation of shadow prices across different industrial sectors shows the necessity of utilizing market power to achieve a cost-effective pollution reduction. According to the shadow price model, there is a negative correlation between the shadow price of SO_2_ emission allowances for the compliance sectors and its proportion coefficient of SO_2_ emissions on the unit production scale. For example, a firm in MCCP has a highest OAWSP of 8.40 ttY/t. The higher shadow prices mean that these sectors’ utility rates for energy are high, and the cost of further energy-saving is relatively high. For instance, some energy-intensive sectors discharge pollutants because of Chinese energy mix. Other energy-intensive sectors release emissions because of inefficiency; hence it may reduce SO_2_ emissions through its own managerial and technological efforts by comparing its shadow price with the market price. In addition, compared national shadow prices with OAWSPs, it’s obvious that the abatement potential of key industries is underestimated considering the provincial differences except for MRPCPPNF. Specifically, the shadow prices of 28 provinces in MWC are from 0.19 to 25.24 ttY/t. Guangxi, Shanxi, and Shaanxi have a higher price in MWC than other provinces. Many provinces have a negative profit on MRPCPPNF in 2012, such as Tianjin, Ningxia, Liaoning, Guangxi, and so on. The shadow prices of MCCP are relatively high at whatever provinces. The MACs of MNMP are from 0.43 to 29.34 ttY/t. The MAC of MPM at the national level is 3.49 ttY/t. The marginal abatement costs in PESE are lower than others sectors in most provinces. PESE and MRPCPPNF should be the focus of the whole China because them have a relatively greater abatement potential.

By comparison, the paper presents the shadow prices (see details in Table 6) from the existing literature and those from this study. Whatever the sulfur dioxide emission trading market in the USA or the carbon emission trading market in EU, electric industry is the first included industry. The existing literatures discuss the shadow prices of coal power plants or coal-burning. In this paper, we regard PESE as the main electric industry. From Table 6, the shadow price of PESE in China is 9136 Yuan/ton, which is lower than estimates from the previous studies in the case of SO_x_. This discrepancy can be explained as follows: different countries and regions have different economic development levels and pollution emissions; our paper estimates shadow prices of industrial sectors from the national, provincial and sectoral levels by the shadow price model combined with the I-O method. Besides, there has been some pilot sulfur dioxide trading programs in China. Jiangsu is the first pilot province and relatively mature. The price of SO_2_ allowances in Jiangsu is 4480 Yuan/ton [35], which is lower than our study. It might indicate that the value of SO_2_ allowances in Jiangsu at the present stage was underestimated.

## 5. Conclusions

China has launched its pilot pollution ETS in order to achieve its target for decreasing national SO_2_ emissions in a cost-effective way. Identifying major SO_2_ emitters and estimating MACs of SO_2_ emissions provides a scientific foundation for the operating rules. This paper quantifies the emission contribution of different industrial sectors in the Chinese economy by an I-O method, and estimates SO_2_ emission allowances’ shadow prices of industrial sectors at the national level, provincial level, and sectoral level by the shadow pricing model. The shadow prices may be interpreted as the MACs of SO_2_ emission allowances for the participating sectors. Conclusions and some important policy implications are summarized as follows.

Firstly, it is suggested that these sectors (MWC, MRPCPPNF, MCCP, MNMP, MPM, and PESE) should be covered in the first stage of pollution ETS. According to the estimation of SO_2_ emissions in different industries at the national level and provincial level, MWC, MRPCPPNF, MCCP, MNMP, MPM and PESE are major emitters. In addition, the PESE sector has the highest share of the whole emissions of SO_2_ at the national level and most provinces (e.g., Jiangsu, Zhejiang, and Henan). Similar to our findings, the SO_2_ ETS in U.S. began in 1995 and covered coal-burning electric utility plants at Phase I [40].

Secondly, the shadow prices of SO_2_ emissions differ at sectoral level. The variation in the estimation of shadow prices shows the necessity of utilizing market power to achieve a cost-effective SO_2_ emission abatement. If an industry has a high shadow price, it will become a buyer in the emission trading market for the gains outweigh the costs of the use of an additional allowance. By contrast, it will reduce its emissions to become a seller in this market with low shadow prices. Through emission trading, both suppliers and buyers would obtain potential benefits or cost savings from the ETS. Thus, the participant industries in the pollution ETS may design appropriate strategies based on our empirical analysis. For example, the heavy industry (e.g., PESE and MRPCPPNF in our calculation) tends to have lower MACs but sufficient SO_2_ emission allowances, and could be better to reserve the remaining allowances because of the underestimation of market prices in pilot markets, like Jiangsu.

Thirdly, the shadow prices of the same sector from different provinces differs a lot. The economic development level and pattern of industrialization and urbanization significantly differ across provinces. For example, the share of heavy industry in the total industry in 2012 was 75.69% in the central region, which is greater than that in the western region (73.30%) and eastern region (70.60%) [10]. Consequently, there are large differences in the SO_2_ shadow prices of the same industrial sector from different provinces. For instance, the shadow prices in MWC are from 0.19 to 25.24 ttY/t. It’s necessary to calculate the shadow prices from different provinces. Thus, it is suggested that at the initial stage of the Chinese pollution ETS, the government should take the provincial differences into consideration to price pollution emission allowances, e.g., there could be some regional trading systems in the first stage of pollution ETS.

In addition, the government may take various measures to keep a perfect ETS (e.g., a fair market). In general, the allowances are freely allocated on “grandfathering” at the first stage of pollution ETS. According to the allocation plan, the light industries that emit less SO_2_ emissions would obtain fewer allowances than the heavy industries. But, our result shows that compared to the firms in light industries, the firms in heavy industries tend to bear lower MACs, e.g., the PESE and MRPCPPNF have the strong incentive to abate pollution to be sellers in the market. According to Zhou et al. [30], it implies that the participating firms in the heavy industries would have an opportunity to invest but the firms in the light industries may have to purchase sufficient allowances, which deviates from the principle of fairness in the market Provincial differences can also lead to similar phenomenon. In this regard, we suggest that the MACs of the participating sectors should be used as a supplementary criterion in the initial allocation of allowances in order to establish a fair pollution trading market.

### Policy Implications

Our empirical results have several important policy and managerial implications which could be considered by the government. Firstly, it is suggested that MWC, MRPCPPNF, MCCP, MNMP, MPM, and PESE should be covered in the first stage of pollution ETS. Secondly, there could be some regional trading systems in the first stage of pollution ETS considering provincial differences. Then, the MACs of participating sectors (or firms) could be used as an important criterion in the initial allocation of SO_2_ allowances. Last but not least, the MACs should be used as a pricing benchmark in the pollution ETS. Our empirical study is remarkably supportive for pollution pricing mechanism of the Chinese pollution ETS. Only when a rational pricing mechanism is established can the pollution ETS create an incentive for polluters to determine the most cost-effective approach to reducing pollution. In addition to the above contributions, this paper calculates the MACs of industrial sectors from three perspectives (national level, provincial level, and sectoral level), which is very flexible in the level of application and can be applied to the cases of sectors, regions, or even the whole country. Thus, it offers an efficient way to track the complex relationship between sectors and provinces and compares the shadow prices of different provinces and sectors in a consistent statistical caliber. Given data availability, this work could be easily extended to other pollution emissions within the same industrial sectors.

## Figures and Tables

**Table 1:** Energy category.

Coals	Gas	Refined Petroleum Products and Coke
Raw Coal	Coke Oven Gas	Coke
Cleaned Coal	Other Gas	Gasoline
Other Washed Coal	Liquid petroleum Gas	Kerosene
Briquettes	Refinery Gas	Diesel Oil
	Crude Oil	Fuel Oil
	Natural Gas	Other Petroleum Products
	Other Coking Products	

**Table 2:** The abbreviations of industries.

Industries	Abbreviations
Mining and Washing of Coal	MWC
Extraction of Crude Petroleum and Natural Gas	ECPNG
Mining of Metal Ores	MMO
Mining and Quarrying of Nonmetallic Mineral and Other Mineral	MQNMOM
Manufacture of Food and Tobacco	MFT
Manufacture of Textiles	MT
Manufacture of Textile Wearing Apparel, Footwear, Leather, Fur, Feather and Its Products	MTWAFLFFIP
Processing of Timbers and Manufacture of Furniture	PTMF
Papermaking, Printing and Manufacture of Articles for Culture, Education and Sports Activities	PPMACESA
Manufacture of Refined Petroleum, Coke Products, Processing of Nuclear Fuel	MRPCPPNF
Manufacture of Chemicals and Chemical Products	MCCP
Manufacture of Nonmetallic Mineral Products	MNMP
Manufacture and Processing of Metals	MPM
Manufacture of Fabricated Metal Products, Except Machinery and Equipment	MFMPEME
Manufacture of General-Purpose Machinery	MGPM
Manufacture of Special-Purpose Machinery	MSPM
Manufacture of Transport Equipment	MTE
Manufacture of Electrical Machinery and Apparatus	MEMA
Manufacture of Communication Equipment, Computer and Other Electronic Equipment	MCECOEE
Manufacture of Measuring Instruments	MMI
Other Manufacture	OM
Scrap and Waste	SW
Repair of Fabricated Metal Products, Machinery and Equipment	RFM
Production and Supply of Electricity and Steam	PSES
Production and Distribution of Gas	PDG
Production and Distribution of Water	PDW
Construction	CN

**Table 3:** National shadow prices of SO_2_ emission allowances in severe polluting industries.

Industries	Shadow Prices ^a^
PSES: Production and Supply of Electricity and Steam	0.91
MRPCPPNF: Manufacture of Refined Petroleum, Coke Products, Processing of Nuclear Fuel	0.20
MWC: Mining and Washing of Coal	3.14
MPM: Manufacture and Processing of Metals	3.49
MNMP: Manufacture of Nonmetallic Mineral Products	4.29
MCCP: Manufacture of Chemicals and Chemical Products	10.09
PDG: Production and Distribution of Gas	2.58
PPMACESA: Papermaking, Printing and Manufacture of Articles for Culture, Education and Sports Activities	19.09

^a^ Unit: ten thousand Yuan/ton (ttY/t).

**Table 4:** The shadow prices of the eight provinces.

	PESE	MRPCPPNF	MWC	MPM	MNMP	MCCP	PPMACESA	CN
Jiangsu								
Shadow price ^a^	0.68	0.73	1.26	5.89	9.4	13.95	8.07	28.22
SO_2_ emission ^b^	401.97	51.40	22.39	73.98	22.39	91.32	22.10	25.77
Emission ratio ^c^	46.84%	5.99%	2.61%	8.62%	2.8%	10.64%	2.57%	3.00%
Zhejiang								
Shadow price ^a^	0.90	— ^d^	— ^d^	5.00	3.34	17.97	11.78	— ^d^
SO_2_ emission ^b^	401.97	— ^d^	— ^d^	25.19	30.81	32.86	10.40	— ^d^
Emission ratio ^c^	57.26%	— ^d^	— ^d^	3.67%	6.34%	6.76%	2.14%	— ^d^
Hunan								
Shadow price ^a^	0.25	−0.41	1.31	5.21	3.16	11.37	11.78	— ^d^
SO_2_ emission ^b^	118.78	23.10	73.28	30.82	39.09	19.68	10.40	— ^d^
Emission ratio ^c^	32.37%	6.29%	19.97%	8.40%	10.65%	5.36%	2.14%	— ^d^
Hubei								
Shadow price ^a^	1.02	−1.25	0.77	3.94	1.61	2.34	— ^d^	25.91
SO_2_ emission ^b^	177.80	10.11	8.71	17.62	82.70	108.21	— ^d^	10.74
Emission ratio ^c^	35.22%	2.00%	1.7%	3.49%	16.38%	21.43%	— ^d^	2.12%
Henan								
Shadow price ^a^	0.10	0.57	1.83	10.65	29.33	11.50	— ^d^	— ^d^
SO_2_ emission ^b^	292.71	68.53	126.42	23.86	21.39	35.81	— ^d^	— ^d^
Emission ratio ^c^	42.58%	9.97%	18.39%	3.5%	3.11%	5.21%	— ^d^	— ^d^
Hebei								
Shadow price ^a^	0.64	0.06	5.51	1.80	2.83	— ^d^	— ^d^	— ^d^
SO_2_ emission ^b^	129.96	190.89	50.25	138.53	37.40	— ^d^	— ^d^	— ^d^
Emission ratio ^c^	15.74%	23.13%	6.08%	16.78%	4.5%	— ^d^	— ^d^	— ^d^
Shanxi								
Shadow price ^a^	0.93	−0.84	21.16	1.38	— ^d^	— ^d^	— ^d^	— ^d^
SO_2_ emission ^b^	36.51	62.61	34.94	16.7	— ^d^	— ^d^	— ^d^	— ^d^
Emission ratio ^c^	5.46%	9.37%	5.23%	2.5%	— ^d^	— ^d^	— ^d^	— ^d^
Shannxi								
Shadow price ^a^	2.44	6.89	25.24	— ^d^	9.41	12.00	— ^d^	28.38
SO_2_ emission ^b^	29.37	31.96	24.47	— ^d^	6.37	5.78	— ^d^	3.57
Emission ratio ^c^	8.87%	9.66%	7.39%	— ^d^	1.92%	1.75%	— ^d^	1.08%

^a^ Unit: ttY/t; ^b^ Unit: ten thousand metric tons; ^c^ The emissions of sulfur dioxide accounted for the entire province are over 1%; ^d^ The missing data is due to the very low emission meaning that the sector is not the polluting emitter in this province or the very low output value meaning that the sector is almost non-existent.

**Table 5:** The shadow prices of China and 28 provinces (Unit: ttY/t).

**Industries**	**National**	**Beijing**	**Fujian**	**Tianjing**	**Ningxia**	**Liaoning**	**Guizhou**	**Guangdong**	**Guangxi**	**Hebei**
MWC	3.14	2.22	0.24	2.20	7.31	1.71	1.42	— ^a^	— ^a^	0.75	
MRPCPPNF	0.20	— ^a^	— ^a^	−0.03	−0.19	−2.92	0.46	2.63	−5.86	0.06	
MCCP	10.09	— ^a^	— ^a^	17.04	0.19	8.29	7.42	— ^a^	5.81	5.51	
MNMP	4.29	— ^a^	— ^a^	14.40	0.62	3.54	0.52	3.86	3.13	2.83	
MPM	3.49	— ^a^	— ^a^	— ^a^	— ^a^	1.29	1.74	9.17	1.00	1.80	
PESE	0.91	— ^a^	15.50	1.32	1.07	0.11	0.93	0.94	0.43	0.64	
**Industries**	**Henan**	**Hainan**	**Heilongjiang**	**Hubei**	**Hunan**	**Jilin**	**Jiangxi**	**Jiangsu**	**Qinghai**	**Xinjiang**
MWC	1.83	— ^a^	0.19	0.77	1.31	1.57	1.00	1.26	0.89	4.19
MRPCPPNF	0.57	6.45	−0.03	−1.25	−0.41	0.21	−0.35	0.73	0.19	−0.27
MCCP	11.50	— ^a^	4.19	2.34	11.37	0.89	17.51	13.95	— ^a^	9.57
MNMP	29.34	4.80	1.40	1.61	3.16	1.93	3.54	9.40	0.43	0.87
MPM	10.65	— ^a^	0.30	3.94	5.21	0.84	5.32	5.89	−0.99	0.64
PESE	0.10	0.52	0.01	1.02	0.25	−0.05	0.64	0.68	0.68	0.45
**Industries**	**Zhejiang**	**Chongqing**	**Shandong**	**Shangxi**	**Shaanxi**	**Shanghai**	**Sichuan**	**Anhui**	**Yunnan**	**OAWSPs ^b^**
MWC	— ^a^	0.55	5.37	21.16	25.24	— ^a^	3.08	7.39	2.74	2.40
MRPCPPNF	— ^a^	1.37	1.54	−0.84	6.89	−8.82	1.33	−1.58	0.18	0.38
MCCP	17.97	6.51	6.67	4.70	12.00	14.89	12.54	10.26	2.68	8.40
MNMP	3.34	3.00	4.77	1.83	9.41	— ^a^	5.53	2.45	1.29	3.89
MPM	5.00	2.40	6.76	1.38	11.46	4.99	1.00	4.26	2.22	2.78
PESE	0.90	0.71	0.39	0.93	2.44	0.65	1.26	1.01	0.93	0.73

^a^ The missing data is due to the very low emission meaning that the sector is not the polluting emitter in this province or the very low output value meaning that the sector is almost non-existent; ^b^ weighted by the SO_2_ emissions’ shares of individual industries from different provinces (Here, negative shadow prices aren’t counted.).

**Table 6:** Comparison between shadow prices of the present and previous studies.

Study	Pollution	Shadow Prices ^a^	Country	Industries
Swinton [21]	$SO_{2}$	18,018	USA	Coal power plants
Färe et al. [27]	$SO_{x}$	13,818	USA	Coal power plants
Mekaroonreung and Johnson [29]	$SO_{x}$	1806	USA	Coal power plants
Coggins and Swinton [36]	$SO_{x}$	2044	USA	Coal-burning
Turner [37]	$SO_{x}$	5782	USA	Coal-burning
Boyd et al. [38]	$SO_{x}$	11,921	USA	Coal-burning
Lee et al. [39]	$SO_{x}$	21,749	Korea	Coal-burning
Tu [28]	$SO_{2}$	20,900	China	The industry industry
Present study	$SO_{2}$	9136	China	PESE

^a^ The unit of shadow price: Yuan/ton ; ^b^ 1 Dollar = 7 RMB.
